# Effects of fiber supplementation on glycemic excursions and incidence of hypoglycemia in children with type 1 diabetes

**DOI:** 10.1186/1687-9856-2014-13

**Published:** 2014-06-21

**Authors:** Nicole Nader, Amy Weaver, Susan Eckert, Aida Lteif

**Affiliations:** 1 Division of Pediatric Endocrinology, Mayo Clinic, 200 1st St SW, Rochester, MN 55905, USA; 2 Biomedical Statistics and Informatics, Mayo Clinic, Rochester, MN 55905, USA; 3 Division of Endocrinology, Mayo Clinic, Rochester, MN 55905, USA

## Abstract

**Background:**

Nutritional therapy is an important component of diabetes management. There is data to suggest that fiber content of foods may affect glycemic response.

**Materials and methods:**

10 children, diagnosed with type 1 diabetes, participated. In the first phase of the study, children followed their usual meal plan. In the second phase, subjects followed the same meal plan except that fiber was added to the diet using a powder supplement (wheat dextrin). Data was collected using a continuous glucose monitoring device. The blood glucose excursion level following each meal was compared between the two phases of the study by fitting a repeated measures regression model. The incidence of hypoglycemia was also compared by fitting a logistic regression model.

**Results:**

There was no difference in the mean blood glucose excursion after meals or the incidence of hypoglycemia between the two phases. There was a strong negative correlation between the amount of fiber supplemented and the mean maximum post-prandial blood sugar after the lunch and breakfast meals (Spearman rank correlation coefficient = −0.86 lunch and −0.76 breakfast).

**Conclusion:**

Our study did not show an overall decrease in glucose excursion or incidence of hypoglycemia with fiber supplementation. We did find a strong negative correlation between the amount of fiber added during the supplemental phase and the mean maximum post-prandial blood sugar after the lunch and breakfast meals. We speculate that different types of fiber may have different effects on blood glucose with wheat dextrin having a greater dampening effect.

## Introduction

Nutritional therapy is an important component of diabetes management. The American Diabetes Association currently recommends that patients with type 1 diabetes monitor their carbohydrate intake either by carbohydrate counting or by the use of exchange diets and match prandial insulin to carbohydrate intake [1]. This method implies that equal portions of carbohydrate have equivalent effects on blood sugar levels. However, there is data to suggest that other factors such as the molecular structure of the carbohydrate and its fiber content result in differential blood glucose responses [2]. High fiber foods as a preferred source of carbohydrate and higher amount of fiber per day are currently recommended [3,4].

Fibers are known to slow down the absorption of carbohydrates after a meal [5].

Despite the fact that children with type 1 diabetes are known to have sub-optimal fiber intake [6,7] and have a tendency towards greater glycemic excursions, there is limited data looking at the effects of a fiber on metabolic control in children. In a cross over study of 23 patients who consumed either a low glycemic diet or a standard diet for two days, it was shown that the low glycemic diet contained more fiber than the standard diet and resulted in lower mean daytime blood glucose values [8]. Rami et al. demonstrated that giving a high-fiber bedtime snack to children with type 1 diabetes flattened the blood glucose curve until midnight [9].

It currently remains unclear what effects a diet rich in fiber would have on diabetes control and glycemic excursions in children with type 1 diabetes. The aim of this study was to determine whether the addition of a fiber supplement to the diet of pediatric patients with type 1 diabetes has an effect on the magnitude of glucose excursions and or the incidence of hypoglycemia. We hypothesized that children with type 1 diabetes receiving a high fiber diet will have lower glucose excursions and a lower incidence of hypoglycemia.

## Research design and methods

### Recruitment

Ten children with type 1 diabetes were recruited from a pediatric endocrinology clinic in Rochester, MN between September 2008 and March 2010. Study staff approached potential participants during their routine diabetes clinic visit and invited them to participate. Inclusion criteria were: diagnosis of type 1 diabetes for at least two years prior to enrollment in the study and 4–16 years of age. Exclusion criteria were: any other associated medical conditions that could potentially affect absorption of nutrients such as celiac disease or inflammatory bowel disease and intercurrent illness during the study period. The study protocol was approved by the Mayo Clinic Institutional Review Board. Study subjects signed assent or consent forms (depending on their age) and both parents signed consent forms prior to participation.

### Study procedure

The study was a prospective interventional observation study with a within-subject cross over design. During the study period the subjects were asked to maintain their usual level of activity. All subjects were asked to present to the outpatient diabetes clinic for an initial study visit. They first met with a dietitian who worked with them on a 3 day meal plan which was representative of their typical daily intake. The subjects were asked to follow that meal plan very closely. They then met with a physician who performed a brief physical exam including an Ears/Nose/Throat, heart, lung, and abdominal exam. Subjects were also asked about the presence of the following signs and symptoms in the preceding 3 days: upper respiratory infection symptoms, vomiting, diarrhea, and fever. If subjects had any signs or symptoms of an infection, upon presentation or during the study period, they were excluded from the study. Subjects were then connected to a continuous blood glucose monitoring device (Medtronic CGMD gold system). Subjects were instructed to continue using their own reflectance meters to check their blood sugars at least four times per day. They were also asked to keep a detailed food diary. Three days later, subjects returned to the clinic and the monitoring device was removed. The study dietitian analyzed the food diary to determine daily caloric intake and fiber intake using the Nutritionist Pro program. Subjects were instructed to contact the study team if they developed any symptoms or signs of infection at any time during the study period.

During the washout period, subjects were asked to lower their insulin dose for unexplained hypoglycemia and increase it for a pattern of hyperglycemia that lasts for three days.

For the second phase of the study which was conducted on similar days of the week, the subjects again returned to the outpatient diabetes clinic and met with a dietitian. There was a two to four week washout period between the study phases. The same meal plan was given except fiber was added to the diet using a powder supplement (Benefiber, fiber supplement, sugar free) in an amount to total 20 grams/1000Kcal/day of fiber. The Benefiber was mixed with beverages and soft foods. The needed total daily amount was divided equally between breakfast, lunch and supper. Subjects again met with a physician, had a brief physical exam performed, and were asked about the presence of symptoms of infection. If subjects had any signs or symptoms of infection, they were excluded from continuing the study. Subjects were then connected to the continuous monitoring device, and given the same instructions as during the first phase of the study. Subjects returned to clinic in three days, at which time the monitoring device was removed.

Data was collected on plasma glucose concentrations using the continuous blood glucose device. The CGMS System Gold provides up to 288 glucose measurements every 24 hours. This data is stored in a Holter-style monitor and can be downloaded into a computer. The system does not display glucose values to the subjects. For each meal, the blood glucose excursion level was determined as the difference between the glucose at the start of the meal and the maximum post prandial glucose within two hours after the meal, but prior to the start of the subsequent meal. Hypoglycemia was defined as a glucose measurement < 70 mg/dL.

### Data analysis

The blood glucose excursion levels following each meal were compared between the two phases (non-fiber and fiber-supplemented) by fitting repeated measures regression models using the MIXED procedure in SAS (version 8.2; SAS Institute, Inc.; Cary, NC). The models were fit after applying the square root transformation to the excursion levels to obtain a more normally distributed outcome. Since each subject had multiple meals over each 3-day phase, the excursion levels were not independent of each other and therefore the appropriate statistical model needed to take into account the correlated nature of the data. To accommodate for this issue and the issue that the multiple meals for each subject were not equally spaced over time, a spatial covariance structure (SP(POW)) was specified. This structure also assumes that measurements closer together are more correlated than measurements obtained further apart in time.

The incidence of hypoglycemia was compared between the two phases (non-fiber and fiber-supplemented) by fitting a logistic regression model using general estimating equation (GEE) methodology available in the GENMOD procedure in SAS. In this model the unit of analysis was an individual glucose measurement. An autoregressive covariance structure was specified to model the correlation between the repeated glucose measurements (i.e. every 5 minutes) over time per subject. This structure assumes that measurements closer together are more correlated than measurements obtained further apart in time.

## Results

A total of 10 subjects (7 females and 3 males) participated in the study. Median age was 11.1 years (range, 8.3-14.2 years). Median duration of diabetes was 4.0 years (range, 2.0-7.3 years). Half of the subjects (5/10) were following a multiple daily injection insulin program and the other half (5/10) were following a continuous subcutaneous insulin infusion program. The amount of fiber added to each subject’s meal plan in order to total 20 grams/1000 kilocalories/day varied from 9–27 (median, 18) grams daily. During the non-fiber-supplemented and fiber-supplemented phases of the study, the subjects recorded a mean of 14.5 and 13.7 meals or snacks, respectively.

The overall median glucose value was determined for each subject during each 3-day phase. During the non-fiber-supplemented phase the overall median glucose level ranged from 89 to 236 mg/dL for the 10 subjects. For 2 of the subjects, the overall median glucose was lower during the fiber-supplemented phase (117 vs 81 mg/dL; 236 vs. 123 mg/dL). However, for the 8 remaining subjects, the overall median glucose level was between 1.5 and 59.3 mg/dL higher during the fiber-supplemented phase.

### Glucose excursions

**Figure 1:**
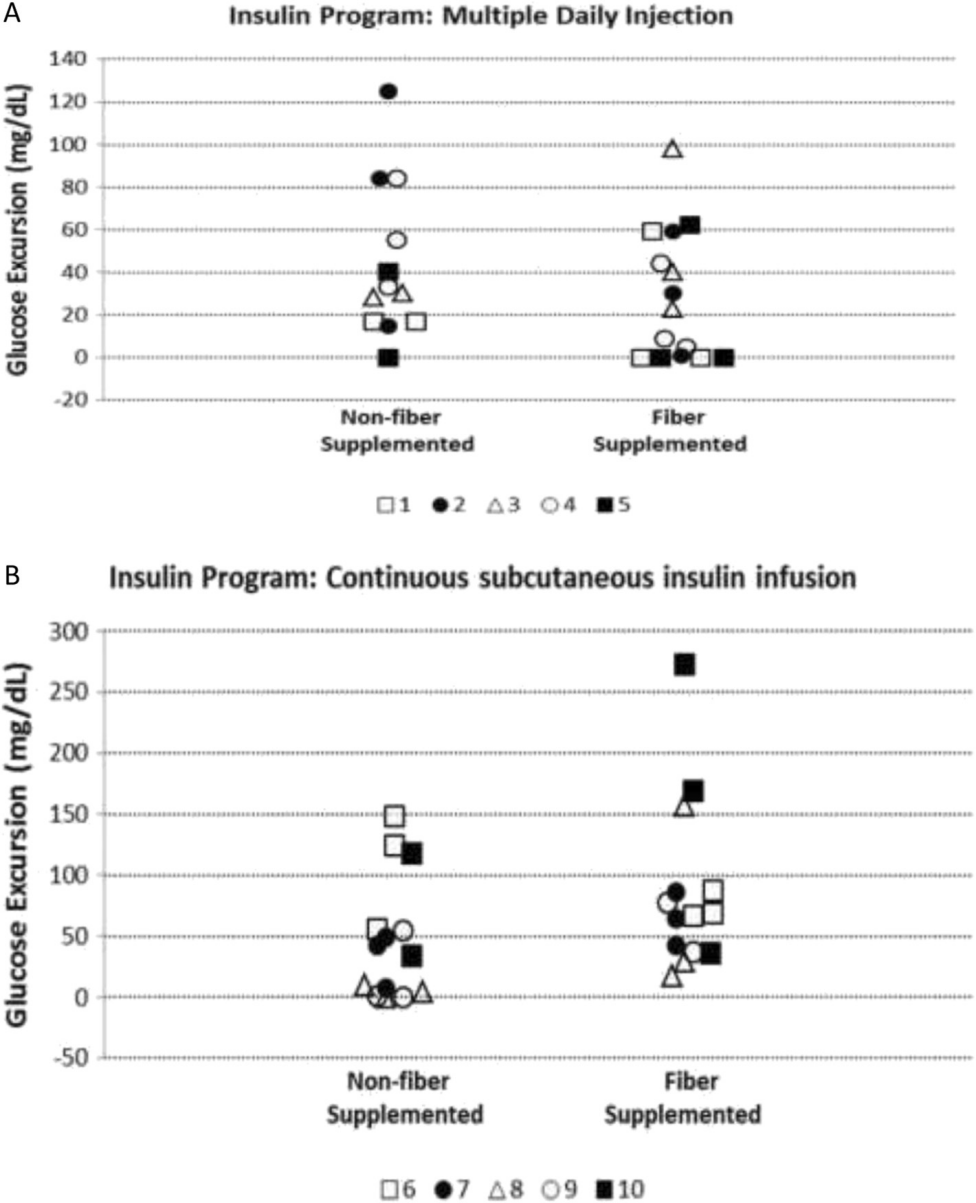
**Blood glucose excursion (maximum post prandial glucose - glucose at the start of the meal) following lunch, separately for the 2 study phases.** During each phase, each such had 2 or 3 lunch meals. **A**. Data for the 5 subjects who followed a multiple daily injection insulin program. **B**. Data of the 5 subjects who followed a continuous subcutaneous insulin infusion program.

**Figure 2:**
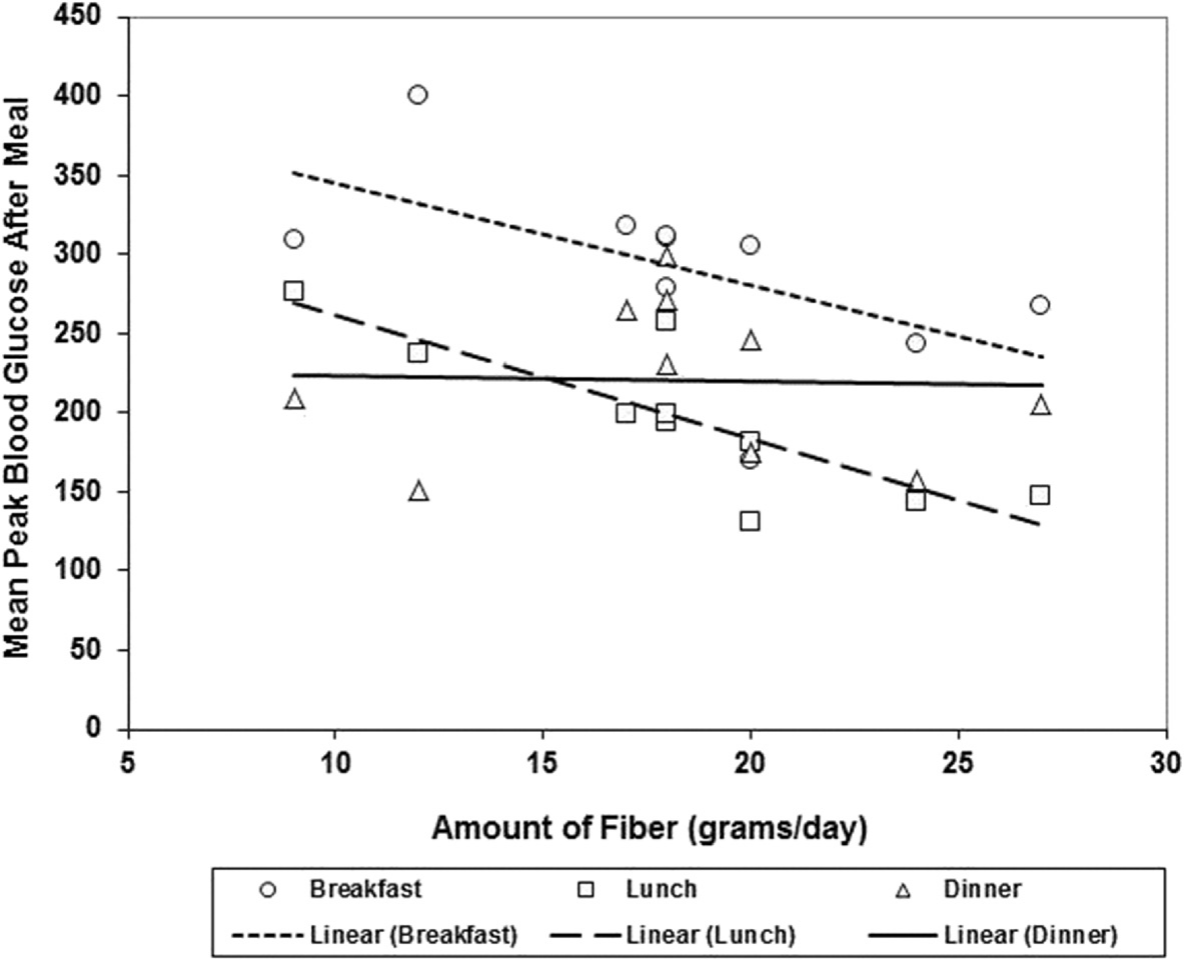
Correlations between the amount of fiber supplemented and the mean peak blood glucose after each meal.

The mean blood glucose excursion (maximum post prandial glucose - glucose at the time of meal) after meals was not significantly different between the non-fiber-supplemented phase of the study and the fiber-supplemented phase (p = 0.17). The results were similar using a model adjusted for the subject’s glucose at the start of each phase (p = 0.18).The mean (SD) glucose excursion after breakfast during the non-fiber-supplemented phase was 114.7 (71.3) vs. 121.2 (49.6) mg/dL during the fiber supplementation phase. For lunch, the mean (SD) glucose excursion during the non- fiber supplemented phase was 47.9 (45) vs. 56.5 (59.8) mg/dL during the fiber supplementation phase (Figure 1A and B). For the evening meal, the mean (SD) glucose excursion during the non-fiber-supplemented phase was 44.7 (34.5) vs. 62.9 (40.2) mg/dL during the fiber supplementation phase.To determine if there was a relationship between the amount of fiber supplemented and glucose excursion, the amount of fiber supplemented per subject was compared with their mean peak blood glucose after the each meal during the fiber supplemented phase. There was not a correlation between the amount of fiber supplemented and the mean maximum blood glucose after the evening meal (Spearman rank correlation coefficient = −0.17). However, there was a strong negative correlation between the amount of fiber added and the mean maximum post-prandial blood sugar after the lunch and breakfast meals, respectively (Spearman rank correlation coefficient = −0.86 for lunch and −0.76 for breakfast, Figure 2).

### Hypoglycemia

During the non-fiber-supplemented phase, the subjects had a total of 8123 glucose measurements, of which 767 (9.4%) were <70 mg/dL. During the fiber phase, the subjects had a total of 8099 glucose measurements, of which 975 (12.4%) were <70 mg/dL. There was no statistical difference in the incidence of hypoglycemia during the two phases of the study (p = 0.55). Half the subjects (5/10) tended to have more hypoglycemia episodes during the non-fiber-supplemented phase (compared to the fiber phase) and the other half tended to have more hypoglycemia episodes during the fiber supplemented phase (Table 1).

**Table 1:** Incidence of hypoglycemia during the two study phases for each of the 10 subjects

**Subject**	**Amount of fiber added (grams)**	**% of glucose measurements < 70 mg/dL**	
		**During the non- supplemented phase**	**During the fiber supplemented phase**
1	27	24.9%	8.2%
2	18	15.5%	11.1%
3	20	8.2%	41.7%
4	18	3.3%	12.3%
5	12	0%	7.6%
6	18	12.0%	8.6%
7	20	6.0%	4.5%
8	17	18.4%	3.1%
9	24	1.8%	3.2%
10	9	2.4%	17.0%
Overall		9.4%	12.4%

The results were also analyzed separately for daytime (8:00 am-7:59 pm) and nighttime (8:00 pm-7:59 am). During the day, 8.5% of blood sugar readings were < 70 mg/dL during the non-fiber supplemented phase vs. 7.5% of measurements during the fiber supplemented phase (p = 0.44). At night, 10.3% of blood glucose measurements were in the hypoglycemic range during the non-fiber supplemented phase vs. 16.3% of measurements during the fiber supplemented phase (p = 0.60).

### Hyperglycemia

During the non-fiber-supplemented phase, the percent of glucose measurements in the hyperglycemic range, defined as blood glucose greater than 180, was 28.0%. During the fiber phase, it was 39%.

## Conclusions

Although our study did not show an overall decrease in glucose excursion or incidence of hypoglycemia with fiber supplementation, we did find a strong negative correlation between the amount of fiber added during the supplemented phase and the mean maximum post-prandial blood sugar after the lunch and breakfast meals.

Dietary sources of fiber vary in composition and include different types of soluble and insoluble fibers. In contrast, the fiber in Benefiber is exclusively wheat dextrin. Wheat dextrin is a soluble fiber that has low viscosity and that is slowly fermented in the large intestine to form short chain fatty acids (SCFAs). A previous study has shown that different types of fibers have differential effects on post prandial blood sugar responses, with pectin administration inhibiting post-prandial glucose rise, but fiber from barley and citrus (Dumovital) having no such effect [10]. Through the production of SCFAs, soluble fibers such as wheat dextrin, can stimulate pancreatic insulin release and may interfere with glycogen breakdown. As a result, blood sugar levels may decrease. Dextrin has been shown to reduce post prandial blood sugars in healthy subjects and reduce fasting blood sugars in type 2 diabetics [11]. We may speculate that wheat dextrin has a greater dampening effect on blood sugar excursion as compared to other types of dietary fiber, so those who added the most wheat dextrin to their diet saw the greatest effects. The effects were only seen after the breakfast and lunch meals. In our patients, breakfast and lunch had higher carbohydrate content. It has been shown that the effect of fibers is most evident in diabetics with more than 55% of their caloric intake coming from carbohydrates [11].

Other studies have shown more significant effects of fiber supplementation on glucose excursion and hypoglycemia. When 60 adult patients with type 1 diabetes were randomized to either a high fiber or low fiber diet for 24 weeks, those in the high fiber diet group had decreased mean daily blood glucose concentrations and fewer hypoglycemic events. Patients who were compliant with the high fiber diet also showed a reduction in hemoglobin A1c [12]. Perhaps, we did not see similar results in our study because the duration of fiber supplementation was significantly shorter. The fermentation of wheat dextrin occurs slowly over 24 hours. Part of the effect of fiber supplements is mediated through the production of SCFAs. We might have seen greater effects if the subjects wore the sensors for greater than 72 hours and if data was analyzed after the first 24 hours. On the other hand, the effect of fiber supplementation has been seen even during very short studies. Monnier et al. showed that the addition of fiber to an oral glucose load improves the pattern of the oral glucose tolerance test by blunting the peak blood glucose, prolonging the time interval to reach the peak blood glucose, and decreasing the rate of blood glucose rise [13].

There are a variety of other factors that influence glucose excursion. These factors include the carbohydrate, fat, and protein contents of the meal, the glycemic index of the food consumed, as well as the cooking methods, processing methods, and the form of foods. Additionally, an individual’s degree of insulin resistance, rate of gastric emptying, and level of physical activity affect blood glucose excursions. Although we asked patients to eat the same types of foods during both phases of the study and we asked them to maintain the same level of physical activity, it is certainly possible that they did not do so and this may have impacted the results. We did review all food records. 6 of 10 participants consumed exactly the same diet at each meal during both study phases. 4 had minor dietary variations but carbohydrate, fat and protein proportions were followed as the subjects were on an exchange type diet with fixed amounts of the different food groups for each meal. A separate analysis was performed. The percent glucose measurement in the hyperglycemic and hypoglycemic range was not significantly different between patients who followed the same exact diet (6/10) and those who did not (4/10).

The strength of this study is the use of the continuous glucose monitoring system, which allowed us to look at data that would otherwise be unavailable using intermittent self-monitoring of blood sugars. However, the limitations include the small sample size and the fact that the meals were not standardized and we relied on patient’s self reported intake. The variability of the insulin doses is also an inherent limitation to the study design. Subjects who were following an exchange type diet received overall a similar insulin dose with each meal. However, and if they had an unexplained low during the washout period, they were asked to decrease their dose. If they had a pattern of three highs, the dose was increased. Subjects using insulin to carbohydrate ratio also adjusted the ratio for unexplained hypoglycemia and a pattern of three high blood sugars. The standardized dose adjustment protocol was outlined by the treating physician. Adjusting the insulin dose for unexplained hypoglycemia was deemed necessary from a safety standpoint.

Based on the results of the current study, there is not enough evidence to recommend fiber supplementation to patients with type 1 diabetes in order to improve glycemic excursions the incidence of hypoglycemia. Future studies, in which all factors including diet, exercise and insulin dose are kept constant, are warranted. Further research in a larger sample size is required. However, all patients should strive to meet dietetic guidelines for the intake of fiber since this level of intake results in very few side effects and may have other benefits.

## Article summary

### Article focus

It currently remains unclear what effects a diet rich in fiber would have on diabetes control and glycemic excursions in children with type 1 diabetes.

The aim of this study was to determine whether the addition of a fiber supplement to the diet of pediatric patients with type 1 diabetes has an effect on the magnitude of glucose excursions and or the incidence of hypoglycemia.

We hypothesized that children with type 1 diabetes receiving a high fiber diet will have lower glucose excursions and a lower incidence of hypoglycemia.

### Key messages

There is no overall decrease in glucose excursion or incidence of hypoglycemia with fiber supplementation in children with type 1 diabetes

There is a negative correlation between the amount of wheat dextrin supplemented and the mean maximum post-prandial blood sugar after the lunch and breakfast meals

### Strengths and limitations

The strength of this study is the use of the continuous glucose monitoring system, which allowed us to look at data that would otherwise be unavailable using intermittent self-monitoring of blood sugars.

However, the limitations include the small sample size and the fact that the meals were not standardized and we relied on patient’s self reported intake.

## Competing interests

The authors declare that they have no competing interests.

## Authors’ contributions

NN designed research, conducted research, and wrote manuscript. SE conducted research. AW performed statistical analysis. AL designed research, wrote manuscript, and had primary responsibility for the final content. All authors read and approved the final manuscript.
